# Supplementary material for: Intensive versus less-intensive antileukemic therapy in older adults with acute myeloid leukemia: A systematic review
Source: PLoS One. 2021 Mar 30;16(3):e0249087. doi: 10.1371/journal.pone.0249087 (PMC8009379; doi:10.1371/journal.pone.0249087)
Supplement: S1 File — MEDLINE search strategy for intensive versus less-intensive antileukemic therapy in older adults with acute myeloid leukemia. (DOCX) [file pone.0249087.s002.docx]

**Supplementary material**

Intensive versus less-intensive antileukemic therapy in older adults with acute myeloid leukemia: a systematic review

**S1. MEDLINE search strategy**

1. exp leukemia, myeloid, acute/

2. acute myeloid leukemia.mp.

3. acute myelogenous leukemia.mp.

4. acute nonlymphocytic leukemia.mp.

5. exp Leukemia, Myelomonocytic, Acute/

6. or/1-5

7. (acut$ or akut$ or agud$ or aigu$).tw,kf,ot.

8. ((promyelocyt$ or promielocitic$ or promyelozyt$ or progranulocyt$) and (leuk?em$ or leuc$)).tw,kf,ot.

9. 7 and 8

10. LEUKEMIA, MYELOID/

11. ACUTE DISEASE/

12. 10 and 11

13. (acut$ or akut$ or agud$ or aigu$).tw,kf,ot.

14. ((myelo$ or mielo$ or nonlympho$ or granulocytic$) and (leuk?em$ or leuc$)).tw,kf,ot.

15. 13 and 14

16. 9 or 12 or 15

17. 6 or 16

18. exp aged/

19. health services for the aged/ or homes for the aged/ or long-term care/ or nursing care/ or exp nursing homes/

20. (advanced years or ageing or aging or elder? or elderly or frail or geriatric? or gerontolog$ or later life or nursing care or nursing home? or old age or oldest old or pensioner? or post-menopausal or postmenopausal or senior or seniors).tw.

21. (aged or aging or ageing or elder$ or geriatric$ or gerontolog$).jw,nw.

22. ('65 year$' or 'over 65' or 'over 70' or 'over 75' or 'over 80' or 'over 85' or '85 year$').tw.

23. or/18-22

24. randomized controlled trial.pt.

25. randomized.mp.

26. placebo.mp.

27. or/24-26

28. Case-Control Studies/ or Control Groups/ or Matched-Pair Analysis/ or ((case* adj5 control*) or (case adj3 comparison*) or control group*).ti,ab.

29. cohort studies/ or longitudinal studies/ or follow-up studies/ or prospective studies/ or retrospective studies/ or cohort.ti,ab. or longitudinal.ti,ab. or prospective.ti,ab. or retrospective.ti,ab.

30. Non-randomi$ed.ab.

31. nonrandomi$ed.ab.

32. (Cohort adj study).ab.

33. (Observational adj study).ab.

34. (Case-control adj study).ab.

35. exp Survival Analysis/

36. or/28-35

37. 27 or 36

38. 17 and 23 and 37
