# Supplementary material for: Intensive versus less-intensive antileukemic therapy in older adults with acute myeloid leukemia: A systematic review
Source: PLoS One. 2021 Mar 30;16(3):e0249087. doi: 10.1371/journal.pone.0249087 (PMC8009379; doi:10.1371/journal.pone.0249087)
Supplement: S2 File — e-Fig 1. All-cause mortality at 30 days after treatment initiation. e-Fig 2. All-cause mortality at 1 year after treatment initiation. e-Fig 3. Proportion of patients who received allogeneic hematopoietic stem cell transplantation. e-Fig 4. Proportion of patients who had serious treatment-emergent adverse events. e-Fig 5. Proportion of patients who had febrile neutropenia. e-Fig 6. Proportion of patients who had anemia. e-Fig 7. Proportion of patients who had neutropenia. e-Fig 8. Proportion of patients who had thrombocytopenia. e-Fig 9. Proportion of patients who had pneumonia. e-Fig 10. Proportion of patients who admitted to intensive care unit (ICU). e-Fig 11. Duration of ICU hospitalization (days). e-Fig 12. Duration of overall hospitalization in days. e-Fig 13. Sensitivity analysis of all-cause mortality assessed with risk of death. (DOCX) [file pone.0249087.s003.docx]

**Supplementary material**

Intensive versus less-intensive antileukemic therapy in older adults with acute myeloid leukemia: a systematic review

**S2. Forest plots**


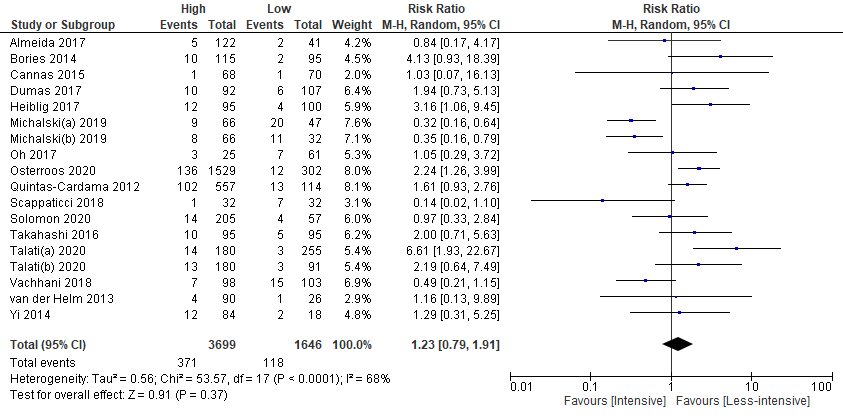


e-Figure 1. All-cause mortality at 30 days after treatment initiation (all from observational studies).


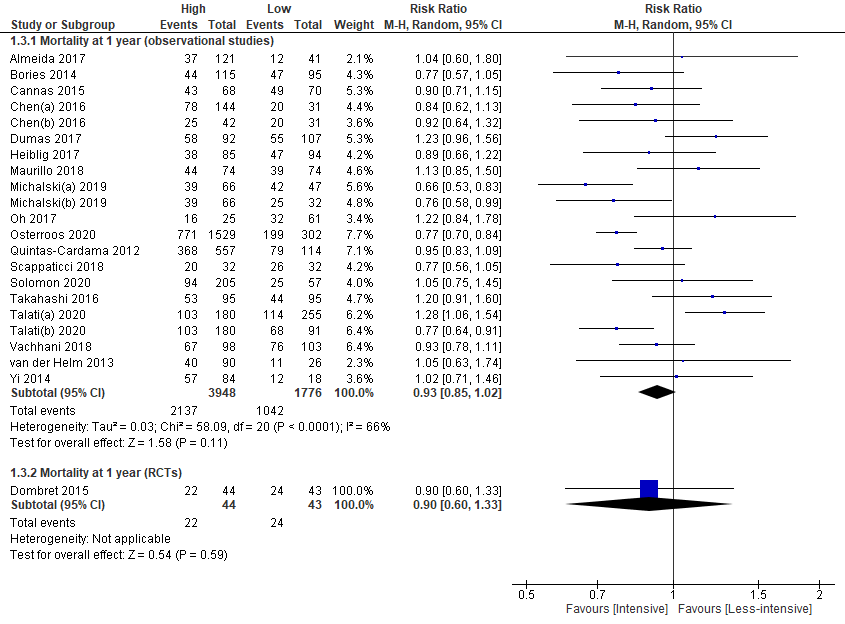


e-Figure 2. All-cause mortality at 1 year after treatment initiation. RCT, randomized controlled trial.


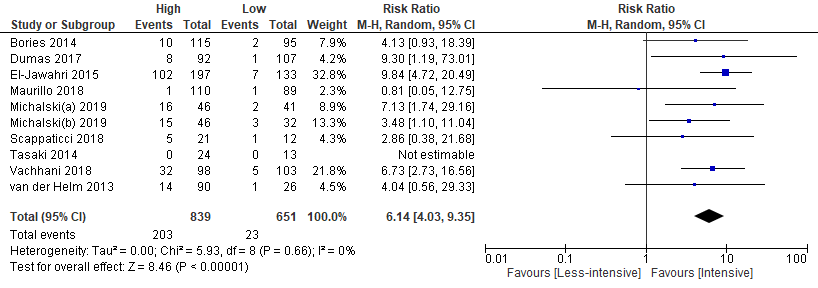


e-Figure 3. Proportion of patients who received allogeneic hematopoietic stem cell transplantation (all from observational studies).


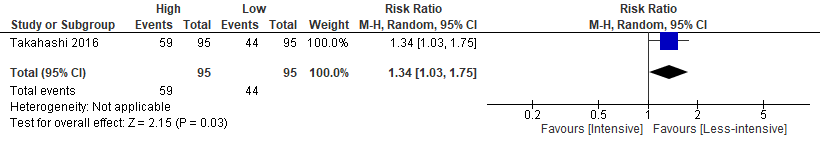


e-Figure 4. Proportion of patients who had serious treatment-emergent adverse events (Grade 3 to 4 severe toxicity, from a single observational study).


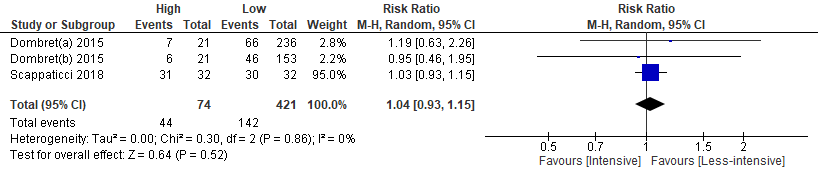


e-Figure 5. Proportion of patients who had febrile neutropenia (all from observational studies).


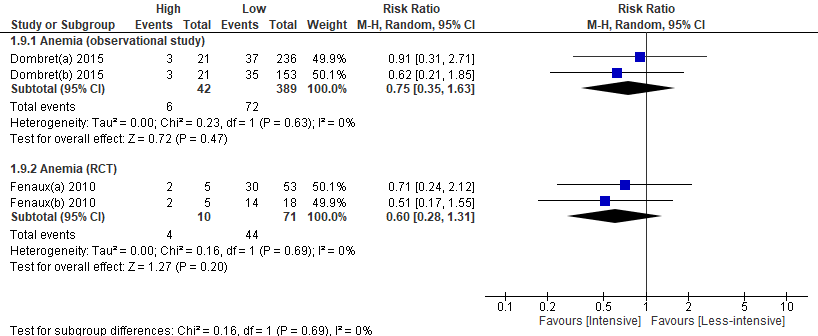


e-Figure 6. Proportion of patients who had anemia.


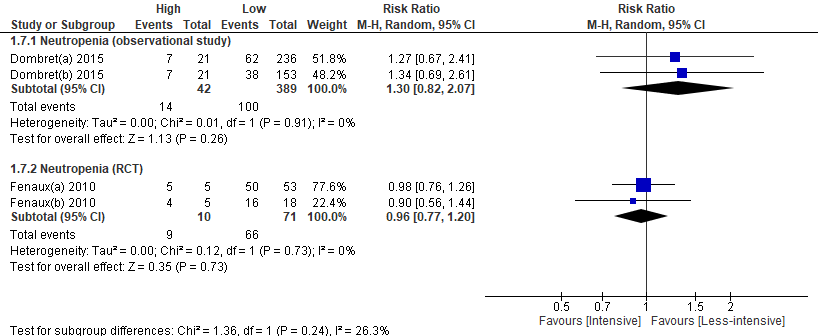


e-Figure 7. Proportion of patients who had neutropenia.


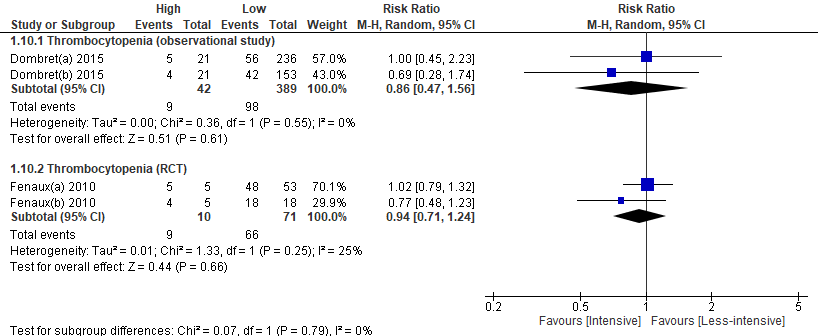


e-Figure 8. Proportion of patients who had thrombocytopenia.


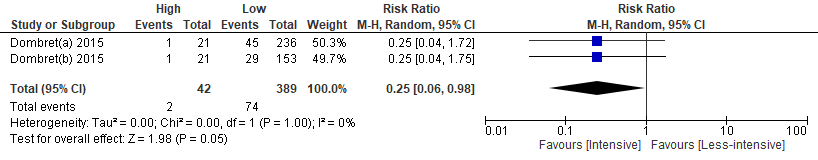


e-Figure 9. Proportion of patients who had pneumonia (from a single study with non-randomized data).


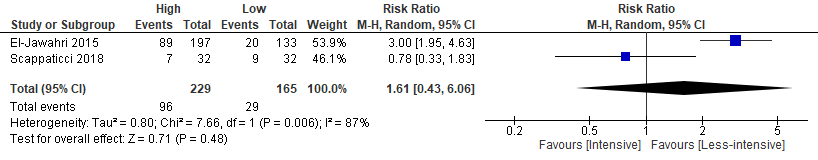


e-Figure 10. Proportion of patients who admitted to intensive care unit (ICU) (from observational studies).


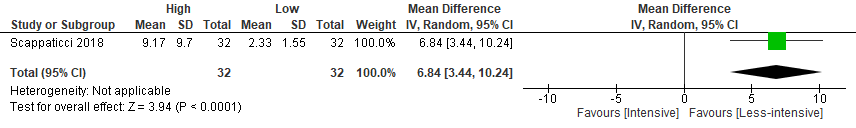


e-Figure 11. Duration of ICU hospitalization (days) (from a single observational study). SD, standard deviation.


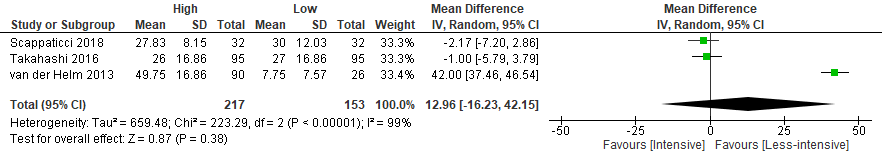


e-Figure 12. Duration of overall hospitalization in days (all from observational studies, SDs in one study (Takahashi 2016) were imputed).


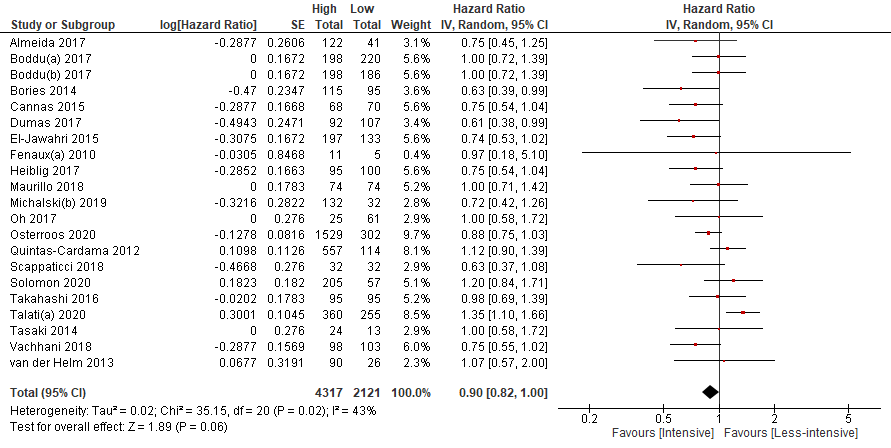


e-Figure 13. Sensitivity analysis of all-cause mortality assessed with risk of death (all from observational studies, HRs and CIs in four studies (Boddu 2017, Maurillo 2018, Oh 2017, and Tasaki 2014) were imputed).
